# International Medical Graduate Physician Deaths From COVID-19 in the United States

**DOI:** 10.1001/jamanetworkopen.2021.13418

**Published:** 2021-06-11

**Authors:** Deendayal Dinakarpandian, Katherine J. Sullivan, Sonoo Thadaney-Israni, John Norcini, Abraham Verghese

**Affiliations:** 1 Department of Medicine, Stanford University, Stanford, California; 2 Department of Medicine, University of Colorado School of Medicine Anschutz Medical Campus, Aurora; 3 Foundation for Advancement of International Medical Education and Research (FAIMER), Philadelphia, Pennsylvania

## Abstract

This case series study examines mortality rates due to COVID-19 among all physicians and international medical graduate physicians in the US.

## Introduction

With more than 26 million confirmed cases of COVID-19 and 400 000 deaths by February 2021, the US has the largest reported disease burden in the world.^1^ Physicians are among the many who have died of this infection. International medical graduates (IMGs) constitute 25% of practicing physicians in the US and often practice in locations and specialties less preferred by US medical graduates (USMGs).^2^ We report on physician mortality from COVID-19, and on mortality of IMGs in particular.

## Methods

This case series study followed the Strengthening the Reporting of Observational Studies in Epidemiology (STROBE) reporting guideline. All analyses used public information related to deceased physicians. Institutional review board approval was not sought based on federal guideline 46.102 (e) (1), which defines human study participants research as involving living individuals, and in compliance with Stanford University’s policy.^3^

Data on deceased US physicians were downloaded on November 23, 2020, from 3 projects tracking health care worker deaths due to COVID-19: MedPage Today (investigative and voluntary reporting; launched April 8, 2020), Medscape (voluntary reporting requiring verifiable information; launched April 1, 2020), and a collaboration between The Guardian and Kaiser Health News (investigation by 70 reporters verifying occupation-related infections; launched April 8, 2020). Obituary and/or news article hyperlinks posted by the 3 projects were researched to verify data (eMethods in the Supplement). Medical school information from DocInfo was used to designate physicians as IMG or USMG.

Data on the numbers of practicing IMGs in different states and specialties published by the American Association of Medical Colleges^4^ were used as a control distribution for comparisons. Risk ratios were calculated to compare observed proportions within the compiled data set with the control distribution. Two-tailed tests were applied to assess the statistical significance of the ratios at a level of *P* ≤ .05. Pearson correlation was used to explore whether IMGs were disproportionately exposed to the pandemic by comparing state-specific IMG proportions with cumulative COVID-19 case counts on May 1, 2020, because most of the deaths took place in May or earlier. Data analyses were performed using R statistical software version 4.0.3 (R Project for Statistical Computing) and SAS statistical software version 9.4 (SAS Institute) from December 2020 to March 2021.

## Results

There was a nonredundant total of 132 physician deaths due to COVID-19 from 28 states; of these physicians, 122 (92%) were male; 33 deaths were reported by The Guardian, 84 deaths were reported by Medscape, and 101 deaths were reported by MedPage Today. IMGs constituted 45% (59 of 132) of the deceased, 1.80 times higher (95% CI, 1.51 to 2.21; *P* < .001) than the 25% national average^2^ of IMGs among practicing physicians. New York, New Jersey, and Florida accounted for 66% of IMG deaths (39 of 59) vs 45% of USMG physician deaths (33 of 73) (Figure). The proportion of IMG deaths within each state was statistically indistinguishable from the corresponding proportion of practicing IMGs, except for New York. However, 67% (89 of 132) of physician deaths occurred in states where the proportion of practicing IMG physicians is greater than the national proportion of 25%. In New York, 60% (24 of 40) of deaths were IMGs, 1.62 times higher (95% CI, 1.26 to 2.09; *P* = .005) than the 37% of practicing physicians in New York who are IMGs.

**Figure.**
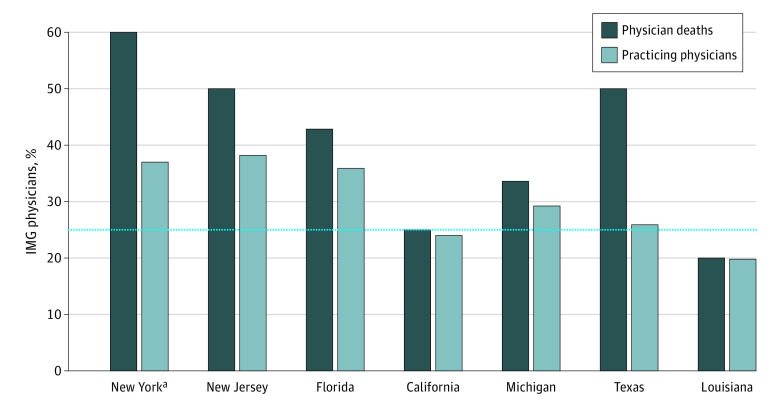
Proportion of International Medical Graduate (IMG) Physician Deaths Due to COVID-19 by State States where at least 5 physicians died are shown. The light bars are percentages of practicing physicians who were IMG within each state, as reported by the American Association of Medical Colleges. The dark bars are the proportions of IMGs among the deceased physicians in each state. The horizontal dotted line denotes the national proportion of practicing IMGs. The numbers of deceased IMGs of the total physician deaths were 24 of 40 for New York, 9 of 18 for New Jersey, 6 of 14 for Florida, 2 of 8 for California, 2 of 6 for Michigan, 3 of 6 for Texas, and 1 of 5 for Louisiana. Counts for states not shown include 4 deaths in Pennsylvania; 3 deaths each in Alabama, Connecticut, Maryland, and Washington; 2 deaths each in Arizona, Illinois, and Indiana; and 1 death each in Arkansas, Georgia, Hawaii, Kentucky, Massachusetts, Minnesota, Missouri, Nevada, Oklahoma, South Carolina, Tennessee, Virginia, and Wisconsin. ^a^New York had a statistically significantly higher proportion of IMG deaths compared with the proportion in practice (60% of deaths [24 of 40] were IMGs, 1.6 times higher [95% CI, 1.26 to 2.09; *P* = .005] than the 37% of practicing physicians in New York who are IMGs).

Among the deceased physicians, 60% (79 of 132) worked in primary care (Table), 1.62 times higher (95% CI, 1.39 to 1.84; *P* < .001) than the national average^4^ of 37% primary care specialists among practicing physicians. Regarding IMGs, 29% (38 of 132) of the deceased physicians were IMGs in primary care, 2.90 times higher (95% CI, 2.13 to 3.65; *P* < .001) than the 10% of all practicing physicians in the US who are primary care IMGs. In contrast, 31% (41 of 132) of the deceased physicians were USMGs who worked in primary care, statistically indistinguishable from the 27% (risk ratio, 1.15; 95% CI, 0.89 to 1.48]; *P* = .33) of all practicing physicians in the US who are primary care USMGs.

**Table.** Physician Deaths From COVID-19 in the US by Specialty

Specialty	Physicians, No. (%)^a^		
	IMG deaths	USMG deaths	Total
All	59 (100)	73 (100)	132 (100)
Primary care^b^	38 (64)	41 (56)	79 (60)
Internal medicine	11 (19)	20 (27)	31 (24)
Family medicine	10 (17)	14 (19)	24 (18)
Pediatrics	10 (17)	2 (3)	12 (9)
Obstetrics and gynecology	6 (10)	4 (5)	10 (8)
Geriatric medicine	1 (2)	1 (1)	2 (2)
Surgery^c^	4 (7)	5 (7)	9 (7)
General surgery	2 (3)	1 (1)	3 (2)
Orthopedic surgery	2 (3)	0	2 (2)
Neurological surgery	0	2 (3)	2 (2)
Oral maxillofacial surgery	0	1 (1)	1 (1)
Reconstructive surgery	0	1 (1)	1 (1)
Psychiatry	3 (5)	5 (7)	8 (6)
Diagnostic radiology	2 (3)	2 (3)	4 (3)
Emergency medicine	2 (3)	2 (3)	4 (3)
Ophthalmology	0	3 (4)	3 (2)
Anesthesiology	1 (2)	1 (1)	2 (2)
Cardiology	1 (2)	1 (1)	2 (2)
Gastroenterology	2 (3)	0	2 (2)
Neurology	1 (2)	1 (1)	2 (2)
Urology	0	2 (3)	2 2)
Unknown	2 (4)	3 (4)	2 (2)
Critical care	0	1 (1)	1 (1)
Dermatology	0	1 (1)	1 (1)
Infectious disease^d^	0	1 (1)	1 (1)
Hematology	1 (2)	0	1 (1)
Neonatology	1 (2)	0	1 (1)
Nephrology	0	1 (1)	1 (1)
Optometry	0	1 (1)	1 (1)
Pathology	0	1 (1)	1 (1)
Podiatry	0	1 (1)	1 (1)
Pulmonology	0	1 (1)	1 (1)
Urgent care	1 (2)	0	1 (1)

Abbreviations: IMG, international medical graduate physician; USMG, US medical graduate physician.

^a^ Percentages may not add to 100% for each column due to rounding.

^b^ Primary care includes internal medicine, family medicine, pediatrics, geriatrics, and obstetrics and gynecology as defined in the American Association of Medical Colleges 2019 State Physician Workforce Data Report.

^c^ Surgery includes general, orthopedic, neurological, oral maxillofacial, and reconstructive surgery.

^d^ Listed as a secondary specialty for a single physician whose primary specialty was internal medicine.

New York and New Jersey accounted for 39% of US cumulative COVID-19 patient cases on May 1, 2020.^5^ These 2 states had the highest proportions of IMGs among practicing physicians (Figure). There was a statistically significant correlation (ρ = 0.66, *P* < .001) between state-specific cumulative COVID-19 cases representing the initial surge in April 2020 and the corresponding state-specific proportions of practicing IMGs,^4^ which persisted even after excluding New York, New Jersey, and Florida (ρ = 0.62, *P* < .001, for 25 remaining states accounting for 45% [60 of 132] of deaths).

## Discussion

In this case series, the proportion of IMGs among physicians (many of whom worked in primary care) who died from COVID-19 was higher than their national proportion among practicing US physicians. A possible reason for this is the observation that the majority of physician deaths occurred in states with relatively larger proportions of IMGs, which were also the states with higher incidence of COVID-19 at the onset of the pandemic. It is also possible that IMGs in a few states (eg, New York) had higher exposure to COVID-19 because of their practice settings; 40% of IMGs work in primary care.^4^ These findings mirror a report from the UK on the high proportion of immigrants among 18 physicians who died of COVID-19.^6^

This study also had some limitations. No definitive or causal conclusions can be reached because of the small numbers, the observational nature of the data set, and the possibility of infections contracted outside clinical practice. The results may not be generalizable to data sets from later stages of the pandemic because of the use of telemedicine and improved measures for physician safety.

The larger number of deaths among IMGs highlights their important contribution to patient care. More research is needed to assess the outcomes for IMGs and USMGs during the COVID-19 pandemic.

## Notes

Supplement. **eMethods.** Data sources
