# Supplementary material for: International Medical Graduate Physician Deaths From COVID-19 in the United States
Source: JAMA Netw Open. 2021 Jun 11;4(6):e2113418. doi: 10.1001/jamanetworkopen.2021.13418 (PMC8196341; doi:10.1001/jamanetworkopen.2021.13418)
Supplement: Supplement. — eMethods. Data sources [file jamanetwopen-e2113418-s001.pdf]

Supplemental Online Content

Dinakarpandian D, Sullivan KJ, Thadaney-Israni S, Norcini J, Verghese A. International medical graduate physician deaths from COVID-19 in the United States. *JAMA Netw Open.* 2021;4(6):e2113418. doi:10.1001/jamanetworkopen.2021.13418

eMethods. Data sources

This supplemental material has been provided by the authors to give readers additional information about their work.

© 2021 Dinakarpandian D et al. *JAMA Network Open.*

eMethods. Data sources

Sources of data on physician deaths due to COVID:

a. Lost on the frontline. The Guardian and Kaiser Health News. 2020. Updated December 2020. Accessed Nov 23, 2020. https://www.theguardian.com/us-news/ng-interactive/2020/aug/11/lost-on-the-frontline-covid-19-coronavirus-us-healthcare-workers-deaths-database.

b. Fiore K. Honoring U.S. Healthcare Workers Who Died from Coronavirus. MedPage Today. 2020. Updated November 4, 2020. Accessed November 23, 2020. https://www.medpagetoday.com/infectiousdisease/covid19/85867.

c. In Memoriam: Healthcare Workers Who Have Died of COVID-19. Medscape. 2020. Updated February 3, 2021. Accessed November 23, 2020. https://www.medscape.com/viewarticle/927976

© 2021 Dinakarpandian D et al. *JAMA Network Open.*
